# Evaluation of the Presence of Native Valvular Disease in Patients With Atrial Fibrillation Using the EHRA (Evaluated Heartvalves, Rheumatic, or Artificial) Classification

**DOI:** 10.1002/clc.70172

**Published:** 2025-07-31

**Authors:** Antonio Escolar Conesa, María Asunción Esteve‐Pastor, Vanessa Roldán, Eva Soler Espejo, José Miguel Rivera‐Caravaca, Pablo Gil Pérez, Eduardo González Lozano, José María Arribas Leal, Sergio Cánovas López, Daniel Saura Espín, María José Oliva Sandoval, Eduardo Pinar Bermúdez, Juan García De Lara, Gregory Y. H. Lip, Francisco Marín

**Affiliations:** ^1^ Department of Cardiology Hospital Comarcal del Noroeste Murcia Spain; ^2^ Department of Cardiology Hospital Clínico Universitario Virgen de la Arrixaca Murcia Spain; ^3^ Instituto Murciano de Investigación Biosanitaria (IMIB‐Arrixaca) University of Murcia Murcia Spain; ^4^ Centro de Investigación Biomédica en Red de Enfermedades Cardiovasculares (CIBERCV) Murcia Spain; ^5^ Department of Hematology Hospital Clínico Universitario Virgen de la Arrixaca Murcia Spain; ^6^ Faculty of Nursing University of Murcia Murcia Spain; ^7^ Department of Clinical Pharmacology Hospital Clínico Universitario Virgen de la Arrixaca Murcia Spain; ^8^ Department of Cardiovascular Surgery Hospital Clínico Universitario Virgen de la Arrixaca Murcia Spain; ^9^ Liverpool Centre for Cardiovascular Science at University of Liverpool, Liverpool John Moores University and Liverpool Heart & Chest Hospital Liverpool UK; ^10^ Department of Clinical Medicine Aalborg University Aalborg Denmark; ^11^ Medical University of Bialystok Bialystok Poland

## Abstract

**Background:**

Atrial fibrillation (AF) in association with native valvular heart disease (VHD) is very common and both entities perpetuate each other due to volume and pressure overload. In 2017, the new EHRA classification (Evaluated Heartvalves, Rheumatic or Artificial) was proposed: EHRA 1 (mechanical prostheses or moderate/severe mitral stenosis), EHRA 2 (native valvular involvement or biological prosthesis) and EHRA 3 (without valve disease). The objective was to analyze the clinical characteristics as well as adverse events in the follow‐up of AF patients under oral anticoagulation classified according EHRA classification.

**Methods:**

A multicenter retrospective observational descriptive study was designed and collected clinical, analytical, echocardiographic characteristics as well as adverse events in the follow‐up of patients with AF who start oral anticoagulation.

**Results:**

1.399 patients were included (mean age 75.3 ± 9.9 years; 659 (47.1%) male), of whom, 63% were classified as EHRA 2. After a median follow‐up of 910 (IQR 730−1018) days, native EHRA 2 patients had higher event rates/patient‐year as well as a higher total rate of adverse events such as cardiovascular mortality (5.5% vs. 1.1% event/patient‐year; 8.7% vs. 1.1% *p* < 0.001) and major adverse cardiovascular events (MACE) (8.9% vs. 3.4% event/patient‐year; 14.2% vs. 3.1% *p* < 0.001), compared with EHRA 3 patients. Multivariate logistic regression analysis showed that native EHRA 2 group was independently associated with all major adverse events.

**Conclusion:**

In anticoagulated AF patients, those with native valve involvement (EHRA 2) have a worse prognosis than patients without valve involvement (EHRA 3). The presence of native valvular disease is shown as an independent risk factor for all‐cause mortality, major bleeding, cardiovascular mortality, ACS, heart failure, and MACE.

## Graphical abstract

In anticoagulated AF patients, those with native valve involvement (EHRA 2) have a worse prognosis than patients without valve involvement (EHRA 3). The presence of native valvular disease is shown as an independent risk factor for adverse outcomes.

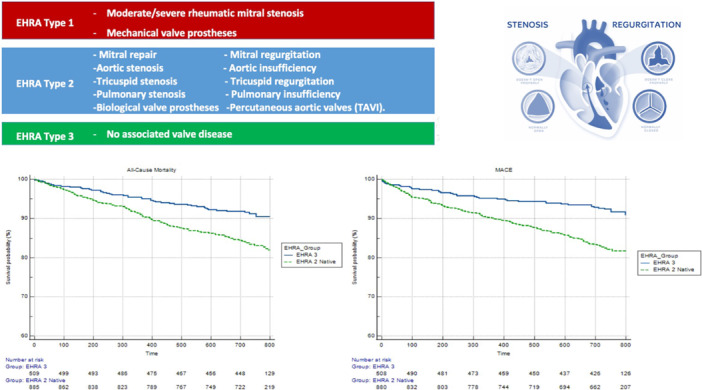

## 1 Introduction

At least one‐third of patients with atrial fibrillation (AF) have some degree of valvular involvement [1, 2]. Like AF, valvular heart disease (VHD) increases with aging and degeneration of cardiac tissues. This degeneration or calcification is accelerated by several risk factors such as hypertension, diabetes mellitus, obesity [3], which are related with both entities.

Long‐term follow‐up data from the original Framingham cohort [4] identified VHD as an independent risk factor for AF development. Both entities perpetuate each other due to the overload of volume and pressure, which causes dilation and remodeling of the cardiac chambers, a mechanism that favors arrhythmia. For example, aortic stenosis increases the systolic pressure of the left ventricle (LV), subsequently, the LV hypertrophies, causing an increase in pressure in the left atrium (LA). Subsequent LA dilates and remodeling. Furthermore, VHD and AF appear to be independent causes of morbidity and mortality when they are related to each other [2, 5, 6, 7, 8, 9].

AF has traditionally been classified as “valvular” and “non‐valvular” [10]. The former referred to patients with AF and moderate or severe mitral stenosis of rheumatic origin and/or mechanical valve prosthesis. The latter involves the rest of non‐rheumatic valve affection [11], or the absence of these. Nevertheless, there is major heterogeneity in the definition of valvular and non‐valvular AF. Some physicians assume that any valve disease should be considered as “valvular” AF. Clinical practice guidelines and clinical trials consider only mechanical valve prosthesis and rheumatic mitral stenosis are considered as “valvular” AF [11]. However, its differentiation in terms of thromboembolism risk has not been investigated in detail and the definition of valvular AF remains controversial.

An appropriate definition of “valvular AF” is mandatory to identify a subgroup of patients with similar pathophysiology of thrombo‐embolism (TE) risk, and treatment strategies; however, this would be challenging given the major heterogeneity of the condition.

In 2017, the European Heart Rhythm Association (EHRA) published a consensus document [12] that proposed subdividing AF into three types according to its valvular involvement, known as the “EHRA (Evaluated Heartvalves, Rheumatic or Artificial) VHD classification.” Type 1 referred to classic valvular AF, that includes moderate/severe rheumatic mitral stenosis and those with mechanical valve prostheses. Type 2 included patients with AF and any other type of valve disease, including those with biological prostheses, TAVR (trans aortic valve replacement) and mitraclip. Type 3 was known as “non‐VHD EHRA” which are those patients with AF without any type of valve abnormalities (Supporting Information S1: Table 1).

Related with this classification, Bisson et al. [13] compared the TE risk (by measuring the incidence of adverse events over time) in patients with AF according to the EHRA classification and to evaluate whether this classification could be more clinically relevant compared to the previous valvular/non‐valvular AF one. They concluded that this classification was useful for categorizing patients in terms of embolic risk and hemorrhagic risk (using the CHA_2_DS_2_‐VASc and HAS‐BLED scores respectively), whereby patients in the second group had a higher embolic risk than patients in the third group. However, in daily clinical practice these types of patients would be anticoagulated based on the CHA_2_DS_2_‐VASc score, despite having a higher embolic risk in studies.

For these reasons, this study aims to evaluate the prevalence of native valvular involvement and its prognostic implications in a cohort of patients with AF in relation to the EHRA types and type of oral anticoagulant (OAC) used (i.e., vitamin K antagonist “VKA” vs. direct OAC “DOAC”).

## 2 Methods

A multicenter retrospective observational descriptive study was designed and collected clinical, analytical, echocardiographic characteristics as well as adverse events in the follow‐up of patients with AF who start OAC (either with VKA or DOACs), both in outpatient clinics and in hospital.

Inclusion criteria were patients with paroxysmal, permanent or persistent AF documented, over 18 years of age and taking OAC (VKA or DOACs) and who have legal capacity to sign informed consent. Exclusion criteria were any type of surgical or percutaneous prosthesis, age < 18 years, those with a formal contraindication for the use of OAC (i.e., uncontrollated major bleeding or frequent falls/frailty), non‐anticoagulated patients with AF, who withdrew their informed consent, patients with active malignancy, clinical or hemodynamic instability, infection active chronic conditions (HIV, HCV, or HBV) or hospital admission in the previous 6 months and patients without possibility of adequate follow‐up.

The clinical and demographic characteristics of all AF patients included in the study will be collected in a Case Report Form (CRF) and classified according to the EHRA types they belonged to.

The study was conducted according to the ethical principles of Declaration of Helsinki and Good Clinical Practice Guidelines and was approved by the Clinical Research Ethics Committee at *Hospital Clínico Universitario Virgen de la Arrixaca (Murcia, Spain*) with the approval number 2018‐9‐1‐HCUVA. The data that support the findings of this study are available from the corresponding author upon reasonable request.

Demographic characteristics were analyzed, as well as data on cardiovascular risk factors and comorbidities. The ischemic risk was calculated using the CHA_2_DS_2_‐VASc score [14]. Haemorrhagic risk was evaluated using the HAS‐BLED [15]. Likewise, transthoracic echocardiography evaluated the presence or absence of native heart valve disease.

After 2‐years follow‐up, major adverse events were collected. TE events are defined as ischemic stroke, transient ischemic attack, acute coronary syndrome and peripheral arterial embolism. We also recorded cardiovascular mortality, all‐cause mortality and major bleeding. Major bleeding events were defined following the International Society of Thrombosis and Haemostasis criteria [16].

We also analysed three combined adverse events: MACE (major adverse cardiovascular events, which included all‐cause mortality, cardiovascular mortality stroke and myocardial infarction/coronary revascularization), Net Clinical Outcome (NCO, including Major bleeding + All‐cause death + ischemic stroke) and the composite outcome of “any thromboembolic event,” that is, myocardial infarction + ischemic stroke + venous thromboembolism.

Continuous quantitative variables are presented as mean (with standard deviation) or median (interquartile range) as appropriate. Categorical variables are presented as a total number and percentage. Descriptive analyses are presented for demographic and clinical variables. Comparisons between categorical variables were carried out using the chi‐square statistic or Fisher's exact statistic as appropriate. The quantitative variables were analyzed using Student's *T* or Mann−Whitney *U* statistical tests as appropriate. The rates of major TE and hemorrhagic events as well as the mortality were analyzed after 2 years of follow‐up. In addition, we used univariate and multivariate Cox logistic regression analysis for identify the predictor variables of adverse events in each of the groups. Statistical analyses were performed using SPSS v19.0 for Windows (SPSS Inc., Chicago, IL, USA), MedCalc v. 16.4.3 (MedCalc Software bvba, Ostend, Belgium), and STATA V 12.0 (Stata Corp., Collage Station, TX, USA). In all analyses, a value of *p* < 0.05 was accepted as statistically significant.

## 3 Results

One thousand three hundred ninty‐nine patients were included in the study (mean age 75.3 ± 9.9 years; 659 (47.1%) male). 886 patients were classified as EHRA 2 (63.3%) with native valve disease and 513 were classified as EHRA 3 (36.7%), without associated valve disease (Table 1). Of the study cohort, 949 patients were anticoagulated with VKA (67.8%) while the other 450 (32.2%) patients with DOACs.

**Table 1:** Baseline characteristics of the AF population.

Variable	EHRA 2 native *n* = 886	EHRA 3 *n* = 513	Total *n* = 1399	*p* value
Age	77.3 ± 8.8	71.85 ± 10.50	75.29 ± 9.86	< 0.001
Male gender	367 (41.4%)	292 (59.9%)	659 (47.1%)	< 0.001
Hypertension	771 (87.1%)	424 (82.6%)	1195 (85.4%)	0.026
Diabetes mellitus	354 (39.9%)	192 (37.4%)	546 (39%)	0.350
Peripheral embolism/PET	25 (2.8%)	13 (2.5%)	38 (2.7%)	0.750
Stroke/TIA	156 (17.6%)	83 (16.2%)	239 (17.1%)	0.490
Coronary disease/MI	193 (21.8%)	67 (13.1%)	260 (18.6%)	< 0.001
PAD	60 (6.8%)	28 (5.5%)	88 (6.3%)	0.195
Vascular disease	223 (25.1%)	84 (16.4%)	307 (22%)	< 0.001
HF or depressed LVEF	291 (32.8%)	88 (17.1%)	379 (27.1%)	< 0.001
Smoker	131 (14.7%)	108 (21.1%)	239 (17.1%)	0.004
Ex‐smoker	53 (5.9%)	38 (7.4%)	91 (5.65%)	
Dyslipidemia	523 (59.1%)	308 (60%)	831 (59.4%)	0.370
Anemia	154 (17.3%)	51 (9.9%)	205 (16.7%)	< 0.001
COPD/OSAS	225 (25.4%)	153 (29.8%)	378 (27%)	0.042
Alcoholism	81 (9.1%)	67 (13.1%)	148 (10.6%)	0.014
Liver disease	52 (5.9%)	30 (5.8%)	82 (5.9%)	0.540
Kidney disease	195 (22.1%)	91 (17.7%)	286 (20.4%)	0.032
Previous bleeding	160 (18.1%)	60 (11.7%)	220 (15.7%)	0.001
Thyroid disease	123 (13.8%)	63 (12.3%)	186 (13.3%)	0.220
Cancer	135 (15.2%)	66 (12.8%)	201 (14.4%)	0.127
Permanent AF	577 (62.8%)	298 (58.1%)	875 (62.5%)	0.005
Paroxysmal AF	309 (34.8%)	215 (41.9%)	309 (22.1%)	
Body mass index (BMI)	30.34 ± 9.9	31.13 ± 9	30.6 ± 9.6	0.040
Glycemia	122 ± 40	121 ± 38	121 ± 39.9	0.238
Creatinine	1.33 ± 4.6	1.23 ± 4	1.30 ± 4.4	0.960
Triglycerides	125 ± 71	136 ± 78	129 ± 74	0.008
HDL	49.7 ± 15	50 ± 16	50 ± 15	0.570
Hemoglobin	13.4 ± 7.6	14 ± 9	13.7 ± 8.3	< 0.001
LVEF	55.2 ± 11	56 ± 11	55.65 ± 11.1	0.071
HAS‐BLED	2.76 ± 1.17	2.43 ± 1.1	2.64 ± 1.2	< 0.001
CHA_2_DS_2_‐VASc	4.4 ± 1.6	3.5 ± 1.6	4.06 ± 1.7	< 0.001

Abbreviations: AF, atrial fibrillation; COPD, chronic obstructive pulmonary disease; EHRA, Eupean Heart Rythm Association; HF, heart failure; LVEF, left ventricular ejection fraction; MI, myocardial infarction; OSAS, obstructive sleep apnea syndrome; PAD, peripheral artery disease PET, pulmonary thromboembolism; TIA, transient ischemic attack.

The study population had a high burden of cardiovascular risk factors, for example, 85.4% had hypertension, 59.4% had dyslipidemia, 39.0% were diabetic and 27.0% had chronic obstructive pulmonary disease/obstructive sleep apnea syndrome (COPD/OSAS). They had an average body mass index (BMI) of 30.64 ± 9.60 kg/m², CHA_2_DS_2_‐VASc score of 4.1 ± 1.7 and HAS‐BLED score of 2.6 ± 1.2. According to the quality of oral anticoagulation in VKA patients, we had only data of INR from 835 (87.9% of VKA patients). The mean of TTR in VKA patients and was 62.1 ± 21.7% and using a cut‐off of TTR > 70% as well‐control, we only observed 329 (39.4%) patients with good quality of OAC with VKA therapy.

According to EHRA type, EHRA 2 patients were older (77.3 vs. 71.8 years; *p* < 0.001) and had higher burden of cardiovascular risk factors like hypertension was more prevalent in EHRA 2 patients (87.1% EHRA 2 vs. 82.6% EHRA 3 *p* = 0.026), coronary artery disease (21.8% EHRA 2 vs. 13.1% ERHA 3, *p* < 0.001) or chronic kidney disease (22.1% EHRA 2 vs. 17.1% EHRA 3, *p* = 0.032) (Table 2).

**Table 2:** Basal chronic therapy in AF population.

Treatment	EHRA 2 Native *n* = 886	EHRA 3 *n* = 513	Total *n* = 1399	*p* value
ACE inhibitors	252 (28.4%)	120 (23.3%)	372 (26.6%)	0.022
ARBs	338 (38.1%)	197 (38.4%)	535 (38.2%)	0.480
Calcium antagonists	330 (37.2%)	154 (30.1%)	484 (34.6%)	0.017
Statins	484 (54.6%)	251 (48.9%)	735 (52.5%)	0.066
Beta blockers	521 (58.8%)	310 (60.4%)	831 (59.4%)	0.290
Digoxin	82 (9.3%)	31 (6.1%)	113 (9.2%)	0.006
Diuretics	465 (52.4%)	225 (43.8%)	690 (49.4%)	0.001
Antidiabetic	265 (29.9%)	131 (25.5%)	396 (28.3%)	0.057
Insulin	71 (8.1%)	32 (6.2%)	103 (7.4%)	0.131
Any antiplatelet agents	224 (25.2%)	90 (17.5%)	314 (22.4%)	< 0.001
Acetylsali acid	135 (15.2%)	53 (10.3%)	188 (13.8%)	0.047
Clopidogrel	35 (3.9%)	18 (3.5%)	53 (3.9%)	
Dual antiplatelet therapy	16 (1.2%)	9 (1.7%)	24 (1.8%)	
Amiodarone	89 (10.1%)	33 (6.4%)	122 (9.9%)	0.003
Flecainide	34 (3.8%)	45 (8.8%)	79 (6.4%)	< 0.001
VKA	594 (67.1%)	355 (69.2%)	949 (67.8%)	0.220
DOAC	292 (32.9%)	158 (30.7%)	450 (32.2%)	
Dabigatran	55 (6.2%)	35 (6.8%)	90 (6.4%)	< 0.001
Rivaroxaban	74 (8.3%)	46 (8.9%)	120 (8.6%)	
Apixaban	119 (13.4%)	59 (11.5%)	178 (12.7%)	
Edoxaban	44 (4.9%)	18 (3.5%)	62 (4.4%)	

Abbreviations: ACE inhibitors, angiotensin‐converting enzyme inhibitors; ARB, angiotensin receptor blocker; DOAC, direct oral anticoagulant; VKA, Vitamina K antagonist.

After a median follow‐up of 910 (730−1018) days it was observed that, in comparison with EHRA 3 patients, EHRA 2 native patients were associated with a higher total rate of adverse events including major bleeding (*p* = 0.001), all‐cause mortality, (*p* < 0.001), cardiovascular mortality (*p* < 0.001) and congestive heart failure (*p* < 0.001), as well as higher rate of composite adverse events (MACE, NCO, or Any TE event) (Table 3).

**Table 3:** Number of events according to EHRA group. The percentage % is with respect to the total. Percentage of event/patient‐year.

Event	EHRA 2 native total (%)	(/100 patient‐years)	EHRA 3 total (%)	(/100 patient‐years)	*p* value
Major bleeding	82 (5.9%)	3.7	22 (1.6%)	1.9	0.001
All‐cause mortality	212 (15.2%)	9.6	59 (4.2%)	3.5	< 0.001
Cardiovascular mortality	122 (8.7%)	5.5	15 (1.1%)	1.1	< 0.001
ACS	46 (3.3%)	2.1	10 (0.7%)	0.7	0.001
Stroke/TIA	67 (4.8%)	3.1	23 (1.6%)	1.1	0.014
HF	324 (23.2%)	14.6	59 (4.2%)	4.6	< 0.001
MACE	198 (14.2%)	8.9	43 (3.1%)	3.4	< 0.001
Net clinical outcome	296 (21.2%)	13.4	91 (6.5%)	7.1	< 0.001
ANY thromboembolic event	114 (8.1%)	5.1	37 (2.6%)	2.9	0.001

Abbreviations: ACS, acute coronary syndrome; ANY thromboembolic event, Myocardial infarction + ischemic stroke + VTE; CHF, congestive heart failure; CV, cardiovascular; MACE, major adverse cardiovascular events. Net Clinical Outcome, major bleeding + all‐cause death + ischmic stroke; TIA, transient ischemic attack.

Based on Cox regression analyses, EHRA 2 patients had 2.21‐fold greater hazard for all‐cause mortality (HR 2.21, 95% CI 1.61−2.92; *p* < 0.001), 2.92‐fold greater hazard for MACE (HR 2.92, 95% CI 2.0−4.0; *p* < 0.001) or 2.31‐folder greater hazard for major bleeding (HR 2.31, 95% CI 1.41−3.71; *p* < 0.001). Higher risk of combined events was also observed, with no differences observed in venous thromboembolism.

**Figure 1:**
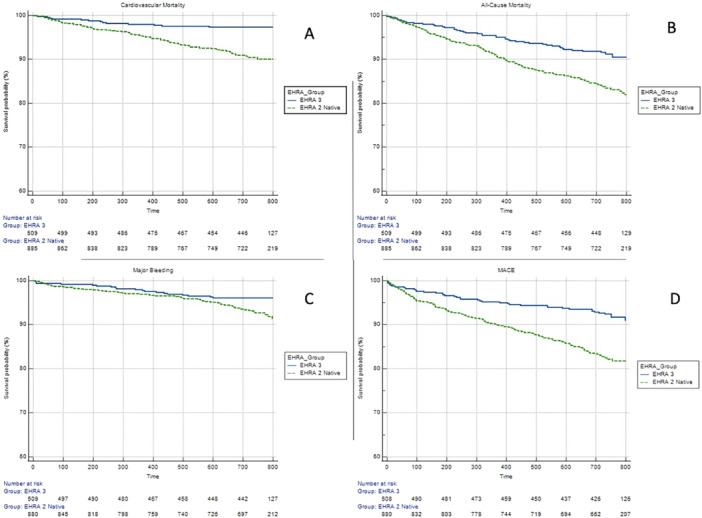
Kaplan−Meier curve. Analysis of survival to the major bleeding (A), all‐cause mortality (B), cardiovascular mortality (C) and MACE event (D). LogRank = 0.001.

Survival analyses by the Kaplan–Meier curves within different EHRA types are shown in Figure 1. EHRA 2 type patients had high risk of all‐cause mortality, cardiovascular mortality, major bleeding and MACE (LogRank = 0.001) (Supporting Information S1: Figures 1−5).

On multivariate Cox logistic regression analysis, EHRA 2 native group was independently associated with the all adverse events, except for stroke/TIA (Tables 4 and 5). Being anticoagulated with a DOAC was protectively associated with the combined MACE event (HR 0.33 95% CI 0.21−0.53; *p* = 0.001).

**Table 4:** Cox regression of adverse events in follow‐up comparing EHRA 2 native vs EHRA 3. The percentage % is with respect to the total.

Event	HR, 95% CI, *p* value
Major bleeding	2.31 (1.41−3.71); *p* < 0.001
All‐cause mortality	2.21 (1.61−2.92); *p* < 0.001
Cardiovascular mortality	5.01 (2.92−8.53); *p* < 0.001
ACS	2.84 (1.40−5.51); *p* < 0.001
Venous thromboembolism	1.05 (0.63−3.62); *p* = 0.920
Stroke/TIA	1.78 (1.10−2.86); *p* = 0.017
HF	3.71 (2.81−4.92); *p* < 0.001
MACE	2.92 (2.00−4.00); *p* < 0.001
Net clinical outcome	2.03 (1.52−2.53): *p* < 0.001
ANY thromboembolic event	1.90 (1.32−2.71); *p* < 0.001

Abbreviations: ACS, acute coronary syndrome; ANY thromboembolic event, Myocardial infarction + ischemic stroke + VTE; CHF, congestive heart failure; CI, Confidence interval; CV, cardiovascular; HR, Hazard Ratio; MACE, major adverse cardiovascular events; Net Clinical Outcome, Major bleeding + All‐cause death + ischmic stroke; TIA, transient ischemic attack.

**Table 5:** Multivariate cox logistic regression with clinical factors related to adverse events.

Condition	HR	CI 95%	*p* value
*Major bleeding*			
EHRA 2 native	1.72	1.03–2.90	0.040
Anemia	2.07	1.27–3.30	0.003
*All‐cause mortality*			
HF or reduced LVEF	1.89	1.45–2.45	< 0.001
Age	1.06	1.05–1.09	< 0.001
Stroke/TIA	1.43	1.04–1.94	0.024
Liver disease	1.76	1.07–2.90	0.025
EHRA 2 native	1.38	1.01–1.93	0.050
*Cardiovascular mortality*			
HF or reduced LVEF	2.09	1.45–3.03	< 0.001
Smoker	2.59	1.28–5.20	0.001
Age	1.08	1.05–1.10	< 0.001
Stroke/TIA	1.67	1.11–2.50	0.014
Alcohol	1.87	1.07–3.20	0.026
EHRA 2 native	3.22	1.80–5.70	< 0.001
*Acute coronary syndrome*			
Coronary disease	3.37	1.77–6.54	< 0.001
Alcohol	1.87	1.07–3.20	0.026
EHRA 2 native	2.34	1.16–4.70	0.017
*Stroke/TIA*			
Age	1.04	1.02–1.92	0.001
Female gender	0.59	0.40–0.85	< 0.001
Stroke/TIA	3.15	2.1–4.5	< 0.001
*Heart failure*			
DOAC	0.49	0.35–0.68	0.001
Age	1.04	1.02 – 1.06	0.001
Kidney disease	1.35	1.05–1.74	0.016
Anemia	1.35	1.04 – 1.7	0.022
EHRA 2 native	2.78	2.07–3.70	< 0.001
*Major adverse cardiovascular event (MACE)*			
DOAC	0.33	0.21–0.53	0.001
Age	1.03	1.02–1.05	0.001
Hypertension	1.72	1.02–2.91	0.039
Stroke/TIA	1.77	1.31–2.40	0.001
HF or reduced LVEF	1.43	1.08–1.89	0.012
EHRA 2 native	2.26	1.58–3.23	0.001
*Net clinical outcome*			
Age	1.05	1.02–1.06	0.001
Stroke/TIA	1.54	1.20–1.98	0.001
Anemia	1.30	1.01–1.68	0.044
HF or reduced LVEF	1.65	1.32–2.06	0.001
EHRA 2 native	1.46	1.15–1.92	0.002
*Any thromboembolic event*			
Hypertension	2.04	1.06–3.90	0.031
Stroke/TIA	2.05	1.42–2.98	0.001
ACS	1.46	1.02–2.11	0.041
EHRA 2 native	1.67	1.12–2.51	0.012

Abbreviations: ANY thromboembolic event, Myocardial infarction + ischemic stroke + VTE; CI, confidence interval; DOAC, direct oral anticoagulant; EHRA, Eupean Heart Rythm Association; HF, heart failure, HR, hazard ratio; LVEF, left ventricular ejection fraction; MACE, major adverse cardiovascular events; Net Clinical Outcome, major bleeding + all‐cause death + ischmic stroke; TIA, transient ischemic attack.

## 4 Discussion

This multicenter cohort study aimed to evaluate the prognostic implications of native valvular disease in patients with non‐valvular AF outside the scope of randomized clinical trials. Our principal findings are as follows: (i) 63% of patients were classified as EHRA 2, showing how common it is to have both AF and valvular heart disease; (ii) the EHRA 2 group had a higher proportion of comorbidities than EHRA 3, and also had a worse prognosis with higher rates of adverse events. The EHRA 2 native group was independently associated with the all‐major adverse events.

According to clinical baseline profile, our study observed that EHRA 2 patients were older, with more comorbidities and had higher score on the hemorrhagic and thrombotic risk scores. However, the EHRA 2 type had higher rates of adverse events, both thrombotic and major bleeding, and it was independently associated with all adverse events after multivariate analysis, apart from stroke.

Our findings contrast with previous studies, like Bisson et al. [13] where the greater TE risk associated with EHRA type 2 VHD status was no longer significant following adjustment for possible confounding risk factors, which was explained by the older age and larger number of comorbidities in the EHRA type 2 VHD group, as reflected by a greater CHA_2_DS_2_‐VASc score. Also, there was a protective effect of the use of DOACs respect to the adverse event: heart failure and the combined event MACE. However, these findings contrast with the study conducted by De Caterina et al. [17] from ENGAGE‐AF trial were although the presence of VHD increased the risk of adverse events, higher‐dose edoxaban regimen had efficacy similar to warfarin in the presence of VHD.

Some AF patients with mild‐moderate native valve disease aortic stenosis, aortic regurgitation, mitral regurgitation, and mild mitral stenosis were included in the pivotal trials (The RE‐LY [18], ROCKET [19], ARISTOTLE [20] and ENGAGE [17] comparing DOACs vs. VKAs). A meta‐analysis of those four phase III AF trials including 13 585 patients showed that high‐dose DOACs provide overall efficacy and safety similar in AF patients with or without VHD [21]

Regarding the use of DOACs in AF patients with native VHD, several studies that supported its effectiveness and safety, but a meta‐analysis published in 2017 concluded that among patients with AF and native VHD, DOACs reduce stroke and systemic embolism compared with warfarin [22]. Evidence shows that apixaban, dabigatran, and edoxaban also reduce bleeding in this patient subgroup, whereas major bleeding (but not intracranial hemorrhage or mortality rate) was significantly increased in VHD patients treated with rivaroxaban. Indeed, a large meta‐analysis of 605 771 real‐world AF patients conducted by Menichelli et al. provided important information about safety and efficacy of DOACs highlighting lower risk of major and gastrointestinal bleeding with apixaban. In our sample, apixaban was the most commonly used DOAC in both EHRA 2 and EHRA 3 patients. One of the main limitation of meta‐analysis is the valve categorize of AF patients included in clinical trials. For experts, the term *nonvalvular* denotes only those patients without mitral valve stenosis or prosthetic heart valves but the heterogeneity of AF patients from trials could be the reason of differences in rate reduction through the four DOACs. Therefore, DOACs could be reasonable alternative to warfarin in AF patients with VHD, as recommended in clinical practice guidelines with Class I, Level of evidence A [23], but not in all VHD patients.

The close relationship between the presence of valvular disease and the appearance of adverse events during follow‐up suggests that the status of valve function should be considered when stratifying risk and planning strategies aimed at improving the prognosis of these patients, therefore they usually require closer monitoring, control of the good quality of anticoagulation, and even switching to DOACs. In the Danish registries, Melgaard et al. [24] completed a retrospective observational registry‐based nationwide cohort study to discern the TE risk related to presence of AF in patients with EHRA Type 2 VHD compared to patients with EHRA type 3 VHD. The main objective of the study was to evaluate whether TE risk assessment can be refined among patients with AF who are younger than 75 years, with EHRA type 2 VHD, and with 0 or 1 CHA_2_DS_2_‐VASc risk score. The authors conclude that in patients typically not recommended OAC for AF, in the presence of Type 2 VHD, the risk may be above the accepted 1% annual TE event rate for initiating a DOAC. This shows the role of type 2 VHD as an independent risk for TE in patients with AF, being consistent with our results in real‐world AF patients.

For that reason, the role of the presence of VHD in AF patients as risk marker is evident. It could be reasonable the use of EHRA type classification in daily clinical practice to identity those high TE and bleeding risk AF patients and to guide OAC therapy.

### 4.1 Limitations

The main limitations of this study derive from its retrospective design. This study did not contain data on VHD severity, which may influence TE risk. Similarly, CHA_2_DS_2_‐VASc and HAS‐BLED scores were calculated at baseline, but risk is not static but dynamic [25] However, the multicenter nature, the number of events recorded and the few losses to follow‐up constitute notable aspects of this study.

## 5 Conclusion

In anticoagulated AF patients, those with native valve involvement (EHRA 2) have a worse prognosis than patients without valve involvement (EHRA 3). The presence of native valvular disease is shown as an independent risk factor for all‐cause mortality, major bleeding, cardiovascular mortality, ACS, heart failure and MACE.

## Author Contributions

A.E.C., M.A.E.P. and F.M. have contributed in analysis, interpretation of data and approval of final publication. M.A.E.P., A.E.C, F.M., V.R., G.Y.L. and J.R.C. have contributed in conception and design. A.E.C., M.A.E.P., F.M., V.R., G.Y.L., J.R.C., P.G.P., E.G.L. and J.M.A.L. have contributed in data acquisition and analysis and interpretation of data. All the authors have contributed in critical revision of publication and approval of final publication.

## Conflicts of Interest

The authors declare no conflicts of interest.

## Supporting information

Supplementary material.

## Data Availability

The authors confirm that the data that support the findings of this study are available from the corresponding author upon reasonable request.
