# Supplementary material for: Evaluation of the Presence of Native Valvular Disease in Patients With Atrial Fibrillation Using the EHRA (Evaluated Heartvalves, Rheumatic, or Artificial) Classification
Source: Clin Cardiol. 2025 Jul 31;48(8):e70172. doi: 10.1002/clc.70172 (PMC12312205; doi:10.1002/clc.70172)
Supplement: Supplementary file 1 — Supplementary material. [file CLC-48-e70172-s001.docx]

**SUPPLEMENTARY MATERIAL**

**Table 1.** Classification of Atrial Fibrillation according to the EHRA Valvular Heart Disease Classification

| **EHRA Type 1** | - Moderate/severe rheumatic mitral stenosis - Mechanical valve prostheses |
| --- | --- |
| **EHRA Type 2** | - Mitral repair - Mitral regurgitation - Aortic stenosis - Aortic insufficiency - Tricuspid stenosis - Tricuspid regurgitation - Pulmonary stenosis - Pulmonary insufficiency - Biological valve prostheses - Percutaneous aortic valves (TAVI). |
| **EHRA Type 3** | - No associated valve disease |


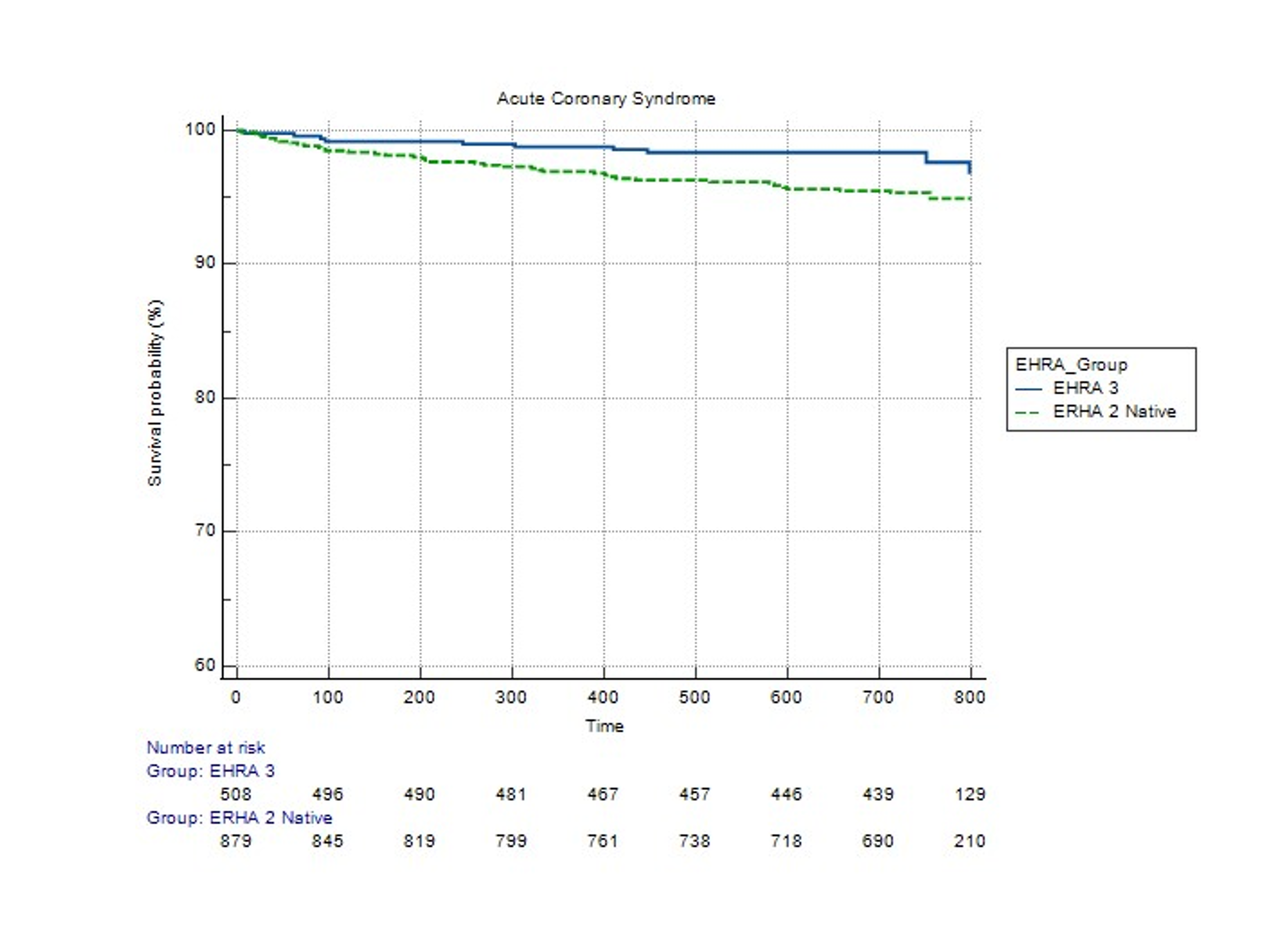
**Supplementary Figure 1.** Kaplan-Meier curve. Analysis of survival to the Acute Coronary Syndrome Event. LogRank=0.002.


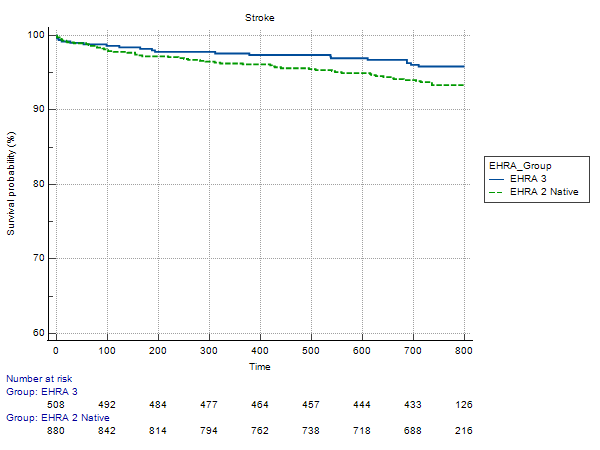
**Supplementary Figure 2** Kaplan-Meier curve. Analysis of survival to the stroke/TIA event. LogRank=0.016.


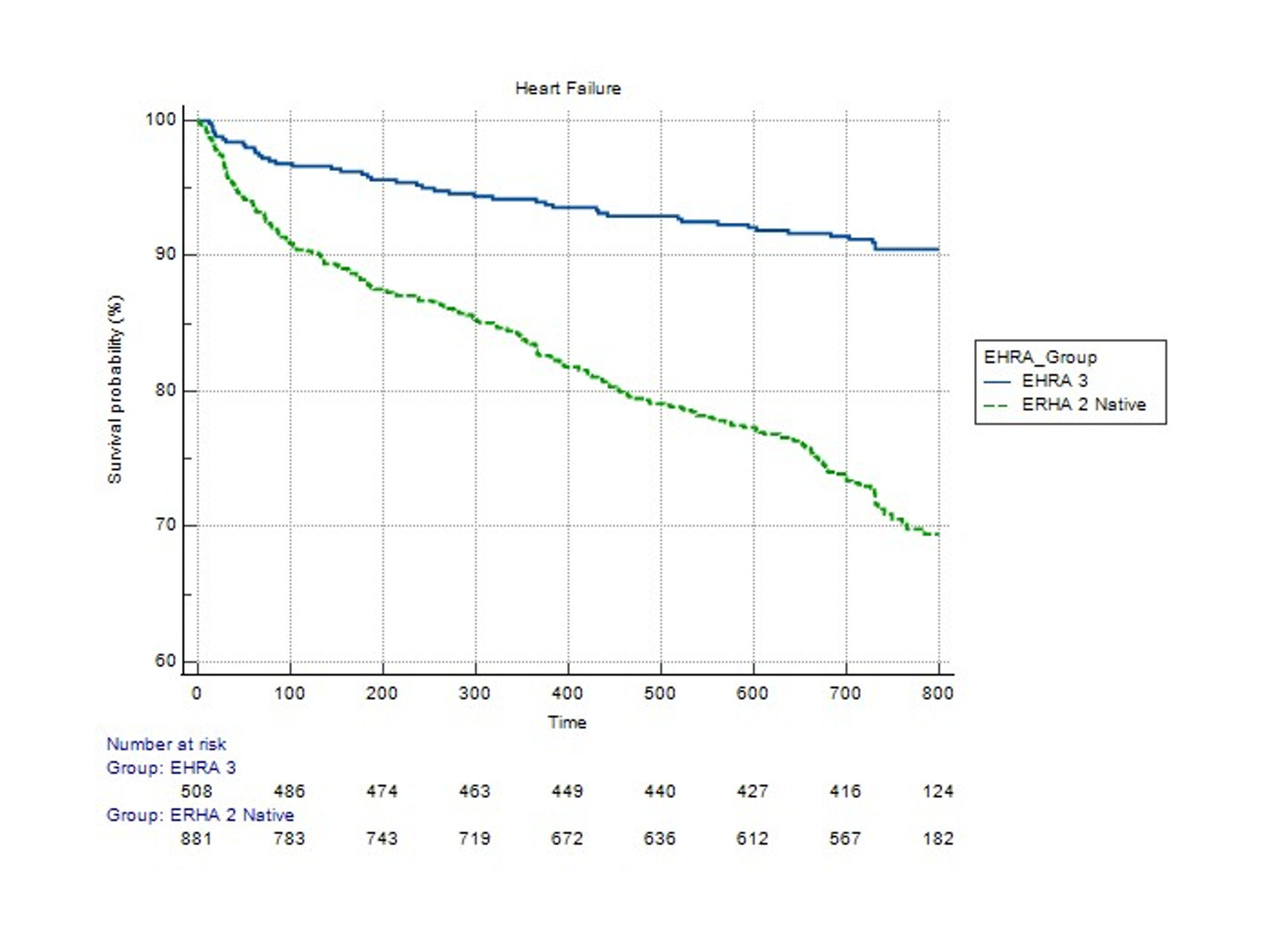
**Supplementary Figure 3** Kaplan-Meier curve. Analysis of survival to the Heart Failure Event. LogRank=0.002.

**Supplementary Figure 4** Kaplan-Meier curve. Analysis of survival to the Net Clinical Outcome event. LogRank=0.001


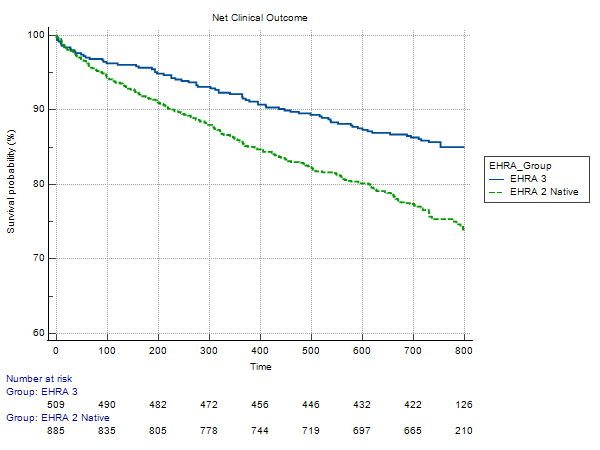


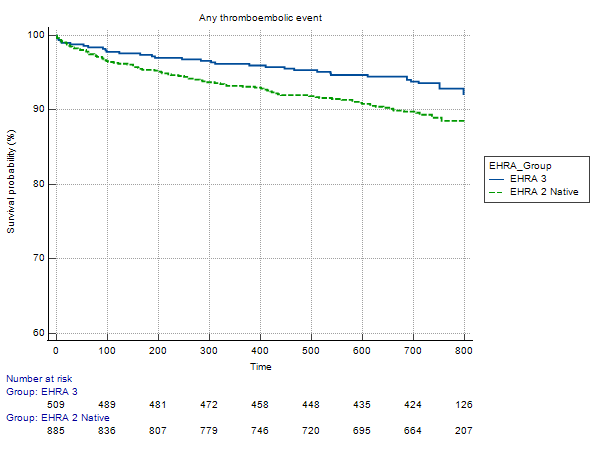
**Supplementary Figure 5** Kaplan-Meier curve. Analysis of survival to “ANY Log Rank=0.001
